# Lexicography of the Horse Disease

**Published:** 1872-11

**Authors:** 


Lexicography of the Horse Disease.—An extensive vocabulary has suddenly arisen in connection with the horse disease. It is variously called Horse influenza, Epihippic, Hippozymotic, Hippozootic, Hippo Grippe, Catarrhal fever, Typhoid Laryngitis, Lung fever, Hippo-malaria, Epyzooty, Equine influenza, Hippic distemper and Equine catarrhal affection. Out of this assortment, Philohippics may select the correct term.—*Med. and Surg. Reporter.*

